# Preliminary species-dependent growth responses of selected oral bacteria to e-cigarette Liquids and aerosol extracts: a single-species in vitro study

**DOI:** 10.1186/s12903-026-10025-x

**Published:** 2026-10-06

**Authors:** Abedelmalek Kalefh Tabnjh, Guglielmo Campus, Sara Alizadehgharib, Anna Lehrkinder, Peter Lingström

**Affiliations:** 1 Department of Cariology, Institute of Odontology, Sahlgrenska Academy, University of Gothenburg, Gothenburg, Sweden; 2 Department of Applied Dental Sciences, Faculty of Applied Medical Sciences, Jordan University of Science and Technology, Irbid, Jordan; 3 Department of Oral Microbiology and Immunology, Institute of Odontology, Sahlgrenska Academy, University of Gothenburg, Gothenburg, Sweden

**Keywords:** Antimicrobial activity, Dental caries, E-cigarette aerosol, Electronic cigarettes, Oral microbiota, *Streptococcus mutans*, Vaping

## Abstract

**Background:**

The increased use of electronic cigarettes (e-cigarettes) has raised concern regarding their potential impact on oral health. E-liquids contain a complex mixture of chemical constituents that may influence the growth of selected oral bacterial species under controlled in vitro conditions. However, comparative data evaluating commercial flavour categories, labelled nicotine concentrations, aerosol extracts, and undiluted e-liquids across both cariogenic and commensal oral bacteria remain limited. This study aimed to provide a preliminary assessment of growth responses of selected oral bacteria representing cariogenic, acid-tolerant, early-colonising, and health-associated species to e-cigarette aerosol extracts and e-liquids with different commercial flavour labels and nicotine concentrations under controlled single-species conditions.

**Methods:**

In this in vitro study, aerosol extracts derived from 18 e-liquids with varying commercial flavour labels and nicotine concentrations (0, 14, and 20–25 mg/mL) were tested against selected oral bacterial species. Two independent bacterial cultures were evaluated, with three analytical replicates per condition within each biological repeat. Single-species planktonic growth was monitored over 48 h, and area under the curve (AUC) values were calculated. Growth-inhibitory activity of aerosol extracts and undiluted e-liquids was evaluated using an agar disk diffusion assay. The pH of undiluted e-liquids was also assessed as a semi-quantitative screening measure.

**Results:**

Descriptively, most AUC means at the product level were below the untreated growth control, with species-dependent magnitudes of change; the largest reduction was observed for *Actinomyces naeslundii* (*A. naeslundii*). No consistent pattern was observed across commercial flavour categories or labelled nicotine concentrations. In the separate *Streptococcus mutans* (S. *mutans)* direct-exposure experiment, mean AUCs were 72.5–82.5% lower than the untreated growth control, and undiluted e-liquids produced zones of growth inhibition in the agar diffusion assay, particularly in flavoured formulations. Approximate pH screening values ranged from 4.0 to 5.0.

**Conclusions:**

These preliminary observations suggest species-dependent growth responses to e-cigarette aerosol extracts and marked growth inhibition by undiluted e-liquids under the tested conditions. Because biological replication was limited (*n* = 2) and the chemical composition and storage stability of the aerosol extracts were not analytically verified, the findings should be interpreted descriptively and require confirmation in larger, chemically characterised, clinically relevant biofilm models.

**Supplementary Information:**

The online version contains supplementary material available at https://doi.org/10.1186/s12903-026-10025-x.

## Introduction

Electronic cigarette (e-cigarette) use has increased substantially in recent years, particularly in Europe and especially among adolescents and young adults. It is now regarded as an emerging public health concern worldwide [1, 2]. Although often perceived as a safer alternative to conventional tobacco smoking, accumulating evidence indicates that e-cigarettes are not without health risks [3]. The oral cavity constitutes the primary site of exposure to e-cigarette aerosols and is therefore particularly susceptible to their potential biological effects [4].

Dental caries, one of the most prevalent chronic diseases globally, is recognised as a biofilm-mediated, non-communicable disease driven by ecological imbalance within the oral microbiota [5–7]. According to the ecological plaque hypothesis, frequent exposure to environmental stressors, such as dietary sugars, may shift the balance of the oral biofilm towards a dysbiotic, acidogenic community, leading to enamel demineralisation and disease progression [6]. This ecological framework is the biological context for the present work, but the experiments provided here assess single-species planktonic growth and agar diffusion instead of evaluating multispecies biofilm ecology.

Among oral microorganisms, *Streptococcus mutans (S. mutans)* is widely recognised as a primary cariogenic species due to its ability to produce acids, tolerance of low pH environments, and ability to form structured biofilms rich in extracellular polymeric substances [8–10]. Other species, including *Streptococcus sobrinus* (S. *sobrinus*) and *Lactobacillus spp*, are also implicated in the development and progression of caries disease [9], whereas commensal streptococci such as *Streptococcus mitis* (*S. mitis*), *Streptococcus sanguinis* (S. *sanguinis*), and *Streptococcus salivarius* (*S. salivarius*) are associated with oral health and microbial homeostasis [9]. The development of dental caries is therefore driven by disruption of this ecological balance rather than by the presence of a single pathogen [11–13].

Electronic cigarette liquids (e-liquids) typically consist of propylene glycol (PG), vegetable glycerine (VG), nicotine, and a wide range of flavouring agents. Upon heating, these components generate aerosols that may contain reactive chemical by-products, including aldehydes and metals [14–18]. Exposure to e-cigarette aerosols may affect the oral cavity through multiple mechanisms. Reported effects include reductions in salivary flow rate, buffering capacity, and pH [19]. E-cigarette exposure may also induce oxidative stress and modulate inflammatory responses in oral cells and tissues, contributing to inflammation and cellular damage [20, 21]. Emerging evidence further suggests that e-cigarette use may influence the composition and function of oral microbial communities with potential differential effects on commensal and cariogenic species [22–24]. In vitro studies have demonstrated that e-cigarette aerosols or liquids may suppress the growth of commensal bacteria while allowing, or even promoting, the persistence of cariogenic species such as *S. mutans*. However, these findings remain inconsistent and appear to depend on product composition, exposure conditions, and experimental models [24–26]. In addition to microbial effects, acidity and other physicochemical properties may also influence the oral environment, but commercial flavour labels and stated nicotine concentrations are crude product categories rather than chemically defined exposure variables. Therefore, chemically characterised, biofilm-based, and clinically relevant studies are needed to make direct causal statements regarding cariogenic dysbiosis or caries risk.

Despite the growing body of research into the oral effects of e-cigarettes, comparative studies simultaneously evaluating multiple commercial formulations, nicotine concentrations, aerosol extracts, and undiluted e-liquids across selected oral bacterial species under standardised experimental conditions remain limited. Most studies conducted so far use simplified exposure systems, and results from single-species cultures cannot by themselves be used to establish changes in community structure, biofilm dysbiosis, enamel demineralisation or clinical disease. Controlled planktonic screening nevertheless provides initial information on direct species-dependent growth responses that can guide subsequent biofilm-based investigation.

Therefore, the aim of this study was to provide an initial, descriptive assessment of the growth responses of selected oral bacterial species to e-cigarette aerosol extracts with different commercial flavour labels and labelled nicotine concentrations in a controlled single-species planktonic in vitro model. A separate exploratory experiment assessed the S. *mutans* response to undiluted e-liquids, and an agar disk diffusion assay was used to screen for local growth-inhibitory activity. The pH of the undiluted e-liquids was also measured as a preliminary screen of their physicochemical properties. The study was not designed to determine population-level formulation effects, dysbiosis of multispecies biofilms, or risk for clinical caries.

## Materials and methods

### E-liquid selection

Commercially available e-liquids were purchased from retail vape shops. All products were formulated with a 50:50 propylene glycol (PG) to vegetable glycerine (VG) base. A total of 18 e-liquids were selected to represent a range of commonly marketed flavour categories, including unflavoured, fruit, mint, tobacco, and dessert flavours. For each flavour category, two or three of the following labelled nicotine (Nic) concentrations were evaluated when available: nicotine-free (0 mg/mL), moderate nicotine (14 mg/mL), and high nicotine (20–25 mg/mL). Detailed information on the tested e-liquids is provided in Table 1. The inclusion of multiple flavour categories, nicotine concentrations, and exposure matrices was intended to enable comparative assessment of species-dependent bacterial responses under standardised in vitro conditions only and should not be interpreted as chemically defined exposure variables.

**Table 1:** Details of the e-liquid products used in this study showing flavour category, nicotine concentration, and country of production

Flavour	Nic-free, Country	Moderate Nic cons, Country	High Nic cons, Country
Cream	0 Nic, UK	14 mg Nic, UK	25 mg Nic USA
Grape&Melon	0 Nic, UK	14 mg Nic, UK	-
Mint	0 Nic, UK	14 mg Nic, UK	-
Strawberry	0 Nic, UK	14 mg Nic, UK	25 mg Nic USA
Tobacco	0 Nic, UK	14 mg Nic, UK	25 mg Nic USA
Unflavoured	0 Nic, SE	14 mg Nic, SE	20 mg Nic SE
Vanilla&Toffee	0 Nic, UK	14 mg Nic, UK	-

### Preparation of e-cigarette aerosol extracts

E-cigarette aerosol extracts were generated using a standardised smoking simulation system (AstraZeneca smoking laboratory, Mölndal, Sweden). Related in vitro studies have used e-cigarette aerosol generated and introduced into aqueous bacterial exposure systems [25, 27]. Aerosols were produced using an electronic nicotine delivery system and captured in phosphate-buffered saline (PBS). This approach was used to enable standardised in vitro exposure to aerosol-derived compounds across different formulations. Briefly, 20 mL of PBS was placed in a collection flask, and aerosol was generated under controlled airflow conditions (0.95 L/min). Puffing parameters were standardised to a 3-second puff duration, with a total of 60 puffs per extraction (equivalent to 3 puffs per mL of e-liquid). Following extraction, samples were aliquoted and stored at − 80 °C until further analysis. Prior to use, extracts were thawed at room temperature and gently mixed on a vortex mixer. However, PBS-based trapping preferentially captures the aqueous, water-soluble fraction of the aerosol and may under-represent volatile or poorly soluble components and buffer acidity. Thus, the extracts should not be considered a complete representation of native aerosol exposure. To reduce the effect of repeated freezing, the extracts were stored in small aliquots and used only once before being discarded. Furthermore, all experiments were conducted within three months of extract preparation.

### Bacterial strains, culture conditions, and growth assay

A panel of selected oral bacterial species, previously collected and stored at the Department of Cariology, was used. Cariogenic species included *S. mutans* (IngBritt) and *S. sobrinus* (OMZ175). Commensal bacteria included *S*. *mitis* (CCUG 31611), S. *sanguinis* (ATCC 10556), S. *salivarius* (CCUG 17825), *Lactobacillus rhamnosus* (*L. rhamnosus*, clinical isolate previously collected, characterised and maintained in the bacterial collection of the Department of Cariology, Institute of Odontology, University of Gothenburg), and *Actinomyces naeslundii* (*A. naeslundii*, ATCC 12104), representing cariogenic, acid-tolerant, early-colonising, and health-associated organisms. *S. mutans* was included in the primary aerosol-extract panel and, as a separate exploratory direct-exposure experiment, was used to evaluate undiluted e-liquids as a model cariogenic species; this experiment was not intended as a cross-species comparison. Bacterial strains were streaked onto and grown on brain heart infusion (BHI) agar plates and incubated at 37 °C in 5% CO₂ overnight. Bacterial suspensions were adjusted to an optical density at 595 nm (OD₅₉₅) of 0.50 before experimentation.

To evaluate the effect of e-cigarette aerosol extracts on bacterial growth, a microtitre plate-based assay was performed. Each well of a sterile 96-well plate contained 50 µL of bacterial suspension, 50 µL of e-cigarette aerosol extract, and 100 µL of BHI broth, resulting in a total reaction volume of 200 µL per well. The following four control conditions were included: (i) untreated growth *control*, bacteria plus BHI without extract; (ii) sterility *blank*, BHI only; (iii) *extract* blank, extract plus BHI without bacteria; and (iv) antimicrobial *positive control*, bacteria plus chlorhexidine. Extract blanks were included to monitor formulation-related absorbance that could arise from colour, turbidity, precipitates, or refractive effects; the values were blank-corrected by subtracting the corresponding formulation-specific extract blank.

Plates were incubated at 37 °C, and bacterial growth was monitored by measuring optical density at 595 nm (OD₅₉₅) every 2 h for 48 h using a microplate reader. Growth curves were generated, and the area under the curve (AUC) was calculated for each condition. The experiment comprised two independent bacterial cultures (biological *n* = 2), with three analytical replicates per condition within each biological repeat (analytical *n* = 3 per repeat), i.e., six well-level measurements in total. The six well-level measurements were used to display within-experiment variability and were not considered six independent biological replicates.

### Kirby–Bauer disk diffusion assay

The antimicrobial activity of e-cigarette extracts and original e-liquids was evaluated using a modified Kirby–Bauer disk diffusion assay. Bacterial suspensions were evenly spread on blood agar plates. Sterile paper disks (6 mm) were impregnated with 10 µL of either e-cigarette aerosol extracts or undiluted e-liquid and placed on the agar surface. Because zone size is influenced not only by biological activity but also by diffusion, especially in viscous PG/VG-based liquids, the assay was regarded as an exploratory screen for local growth inhibition rather than proof of bacterial killing.

In addition to the primary growth-curve panel, two additional cariogenic species were included in an exploratory disk diffusion panel: Bifidobacterium dentinum (B. dentinum, CCUG 18367) and a Lactobacillus (clinical isolate). Disks treated with 0.2% chlorhexidine served as the antimicrobial positive controls. Plates were incubated at 37 °C in 5% CO₂ for 48 h. Zones of inhibition were measured in millimetres, excluding the disk diameter. All experiments were performed in duplicate on the same agar plate.

### pH measurement

The pH of undiluted e-liquids was assessed semi-quantitatively using pH indicator strips, with readings recorded after 1 min of exposure for comparative screening. Titratable acidity and the pH of the final bacterial exposure mixtures were not measured. Because PBS buffers acidity, the pH of aerosol extracts was not assessed.

### Statistical analysis

Bacterial growth was summarised using area-under-the-curve (AUC) values derived from growth curves over the 0–48 h period using the trapezoidal rule. The study had an exploratory design comprising two independent bacterial cultures (biological *n* = 2), with three analytical replicates per condition within each biological experiment. Analytical replicates were used to characterise within-experiment measurement variability and were not treated as independent biological observations. Because the number of independent biological experiments was insufficient for robust estimation of between-experiment biological variability, no inferential statistical testing was used to support comparisons among conditions. Results are therefore presented descriptively using the observed AUC values, means, ranges, and percentage changes relative to the untreated growth control, as appropriate. All findings should be interpreted as preliminary observations specific to the bacterial strains, products, and experimental conditions evaluated.

## Results

### Bacterial growth

**Fig. 1:**
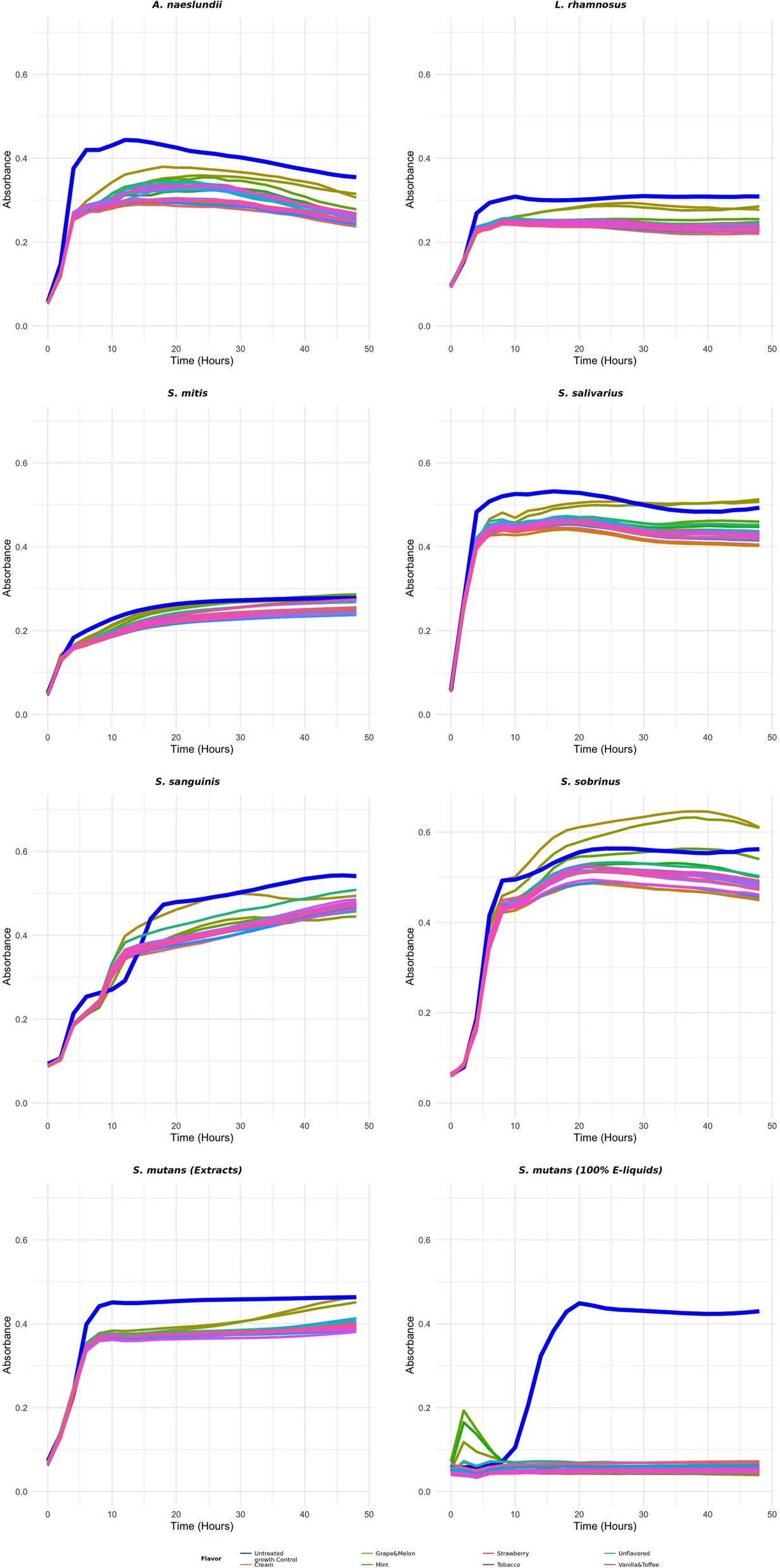
Growth curves following exposure to e-cigarette aerosol extracts, for selected oral bacterial species: *Actinomyces naeslundii* (*A. naeslundii*), *Lactobacillus rhamnosus* (*L. rhamnosus*), *Streptococcus mitis* (*S. mitis*), *Streptococcus salivarius* (*S. salivarius*), *Streptococcus sanguinis* (*S. sanguinis*), *Streptococcus sobrinus* (*S. sobrinus*), *Streptococcus mutans* (*S. mutans*) exposed to aerosol extracts, and *S. mutans* in the separate exploratory undiluted e-liquid experiment. The blue curve represents the untreated growth control

Across the aerosol-extract experiments, most product-level mean AUCs were lower than the untreated growth control. The magnitude and direction of change in the AUC values varied by species and formulation (Fig. 1; Supp. Table 1). Descriptive mean AUC reductions for *L. rhamnosus*, compared to the untreated growth control, ranged from 9.2% to 23.4%, for *S. salivarius* from 2.6% to 16.7%, for *S. sanguinis* from 6.4% to 15.8%, for *S. mitis* from 0.8% to 16.0%, for *A. naeslundii* from 13.5% to 31.9%, and for *S. mutans* from 10.3% to 18.7% across the aerosol-extract formulations. For *S. sobrinus*, changes ranged from a 14.7% decrease to a 10.1% increase, indicating that not all formulations resulted in reduced mean growth. In the separate undiluted e-liquid experiment, mean S. mutans AUC values were 72.5% to 82.5% lower than the untreated growth control.

**Fig. 2:**
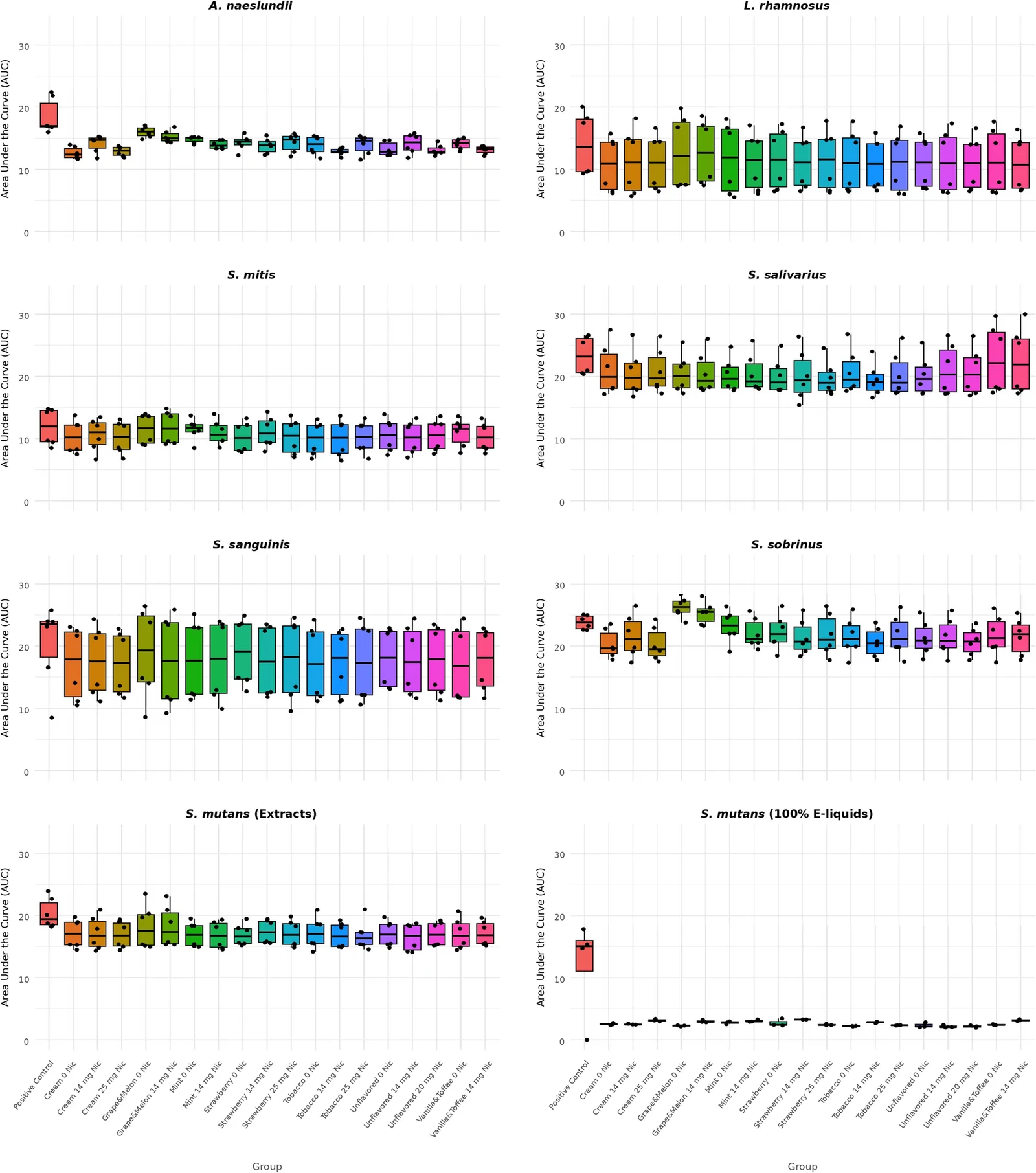
Descriptive boxplots showing AUC distributions across the untreated growth control and individual commercial formulations for *Actinomyces naeslundii (A. naeslundii)*,* Lactobacillus rhamnosus (L. rhamnosus)*,* Streptococcus mitis (S. mitis)*,* Streptococcus salivarius (S. salivarius)*,* Streptococcus sanguinis (S. sanguinis)*,* Streptococcus sobrinus (S. sobrinus)*,* Streptococcus mutans (S. mutans)* exposed to aerosol extracts, and *S. mutans* exposed to different flavours of undiluted e-liquids (100%). The six well-level observations per condition represent three analytical replicates within each of two biological repeats and should not be interpreted as six independent biological observations

The magnitude of the descriptive AUC change differed substantially among bacterial species, indicating species-dependent responses to e-cigarette aerosol exposure. The most pronounced reduction in AUC was observed for *A*. *naeslundii*, whereas moderate changes were detected for *L. rhamnosus*,* S. mutans*,* S. mitis*,* S. sanguinis*,* and S. salivarius* (Fig. 2 and Supp. Figure 1).

**Fig. 3:**
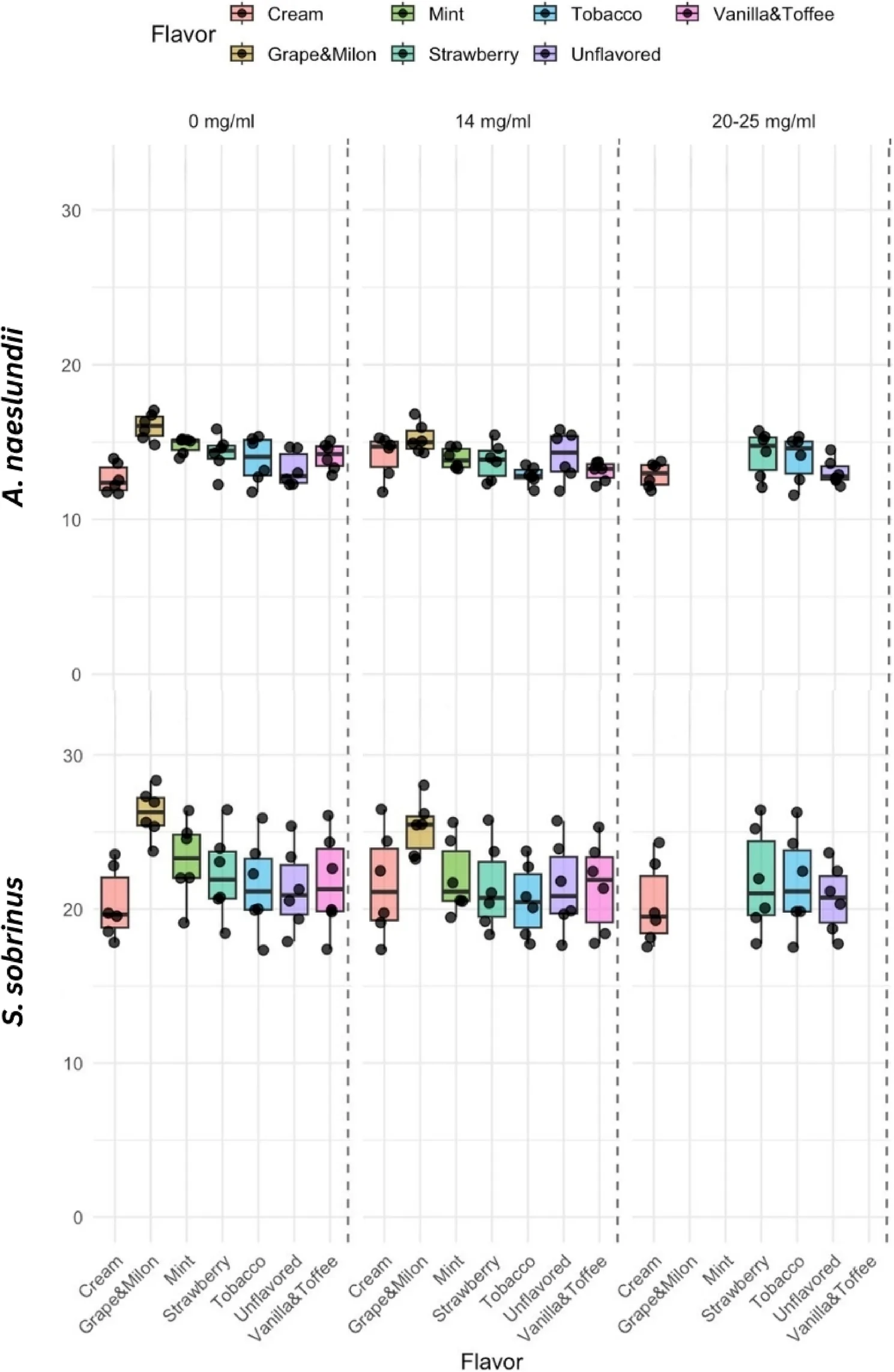
Descriptive boxplots showing AUC distributions across commercial flavour groups stratified by labelled nicotine concentration for *Actinomyces naeslundii* (*A. naeslundii*) and *Streptococcus sobrinus* (*S. sobrinus*). The figure presents a descriptive visualisation of the observed well-level measurements. No inferential statistical testing or population-level flavour or nicotine effect is inferred because biological replication was limited to *n* = 2

There was descriptive variation among commercial flavour groups for *A. naeslundii* and *S. sobrinus*; however, given the limited biological replication, these observations were not subjected to inferential statistical testing and no population-level flavour effect could be inferred (Fig. 3).

When e-cigarette aerosol extracts were grouped according to labelled nicotine concentration (0 mg/mL, 14 mg/mL, and 20–25 mg/mL), no consistent descriptive pattern in bacterial growth was observed across all species in the reported analysis (Supp. Figure 2).

In contrast, direct exposure of S. mutans to undiluted e-liquids resulted in a marked descriptive reduction in AUC values compared with the untreated growth control. Under these conditions, the 14 mg/mL nicotine group exhibited numerically less growth inhibition compared with the other labelled nicotine groups.

### Kirby–Bauer disk diffusion assay

Disk diffusion assays showed no measurable zones of inhibition around disks when bacterial strains were exposed to e-cigarette aerosol extracts, indicating the absence of detectable growth-inhibitory activity under these conditions. In contrast, direct application of undiluted e-liquids to the disks resulted in clear zones of inhibition for several bacterial species. The extent of growth inhibition varied depending on the e-liquid formulation.

**Fig. 4:**
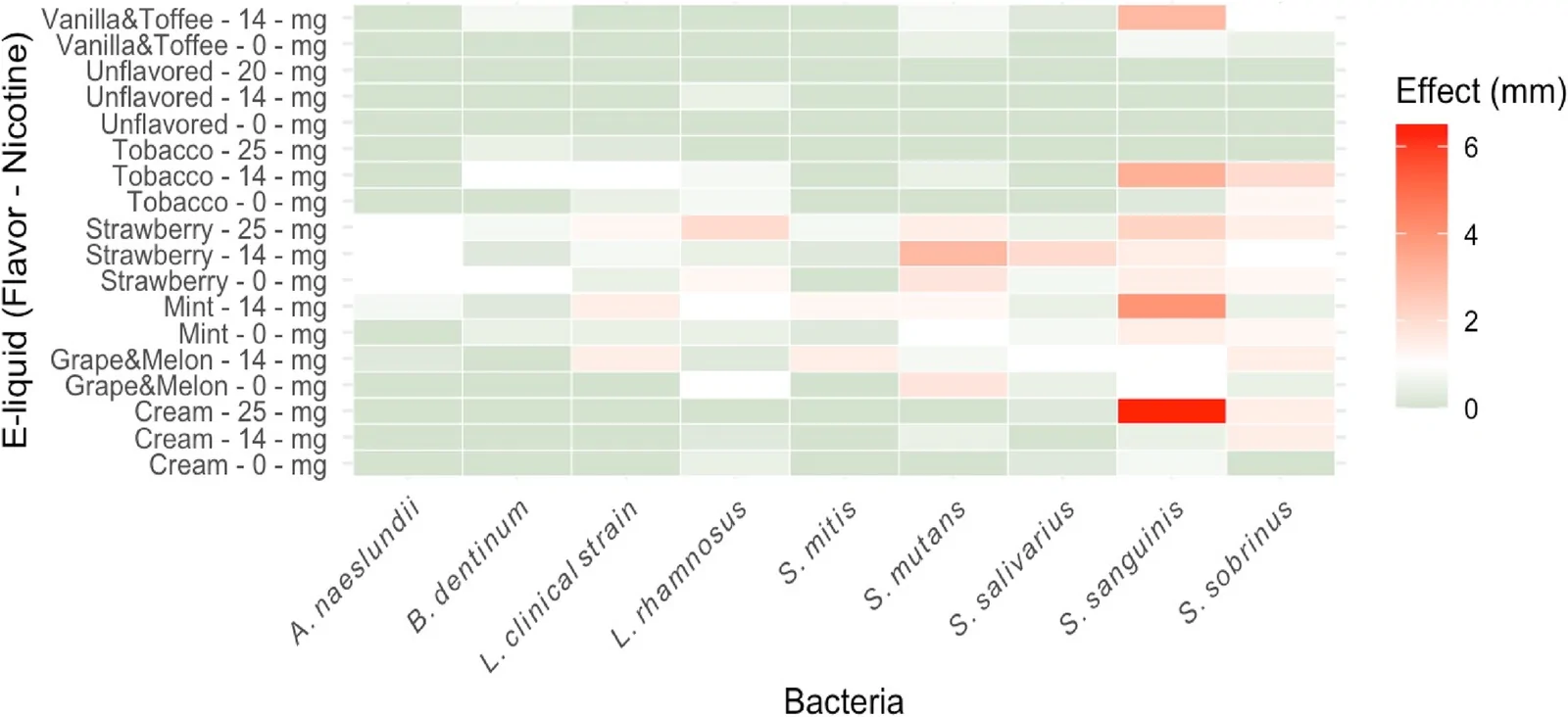
Heatmap of inhibition-zone diameters observed after direct application of undiluted e-liquids in the exploratory agar diffusion assay. *B. dentinum* and the Lactobacillus clinical isolate were included only in the additional exploratory panel. The heatmap represents zones of growth inhibition

Flavoured e-liquids generally exhibited larger zones of growth inhibition than unflavoured formulations, whereas unflavoured e-liquids showed minimal or no inhibitory activity regardless of nicotine concentration (Fig. 4, Supp. Table 2).

### pH measurement

**Fig. 5:**
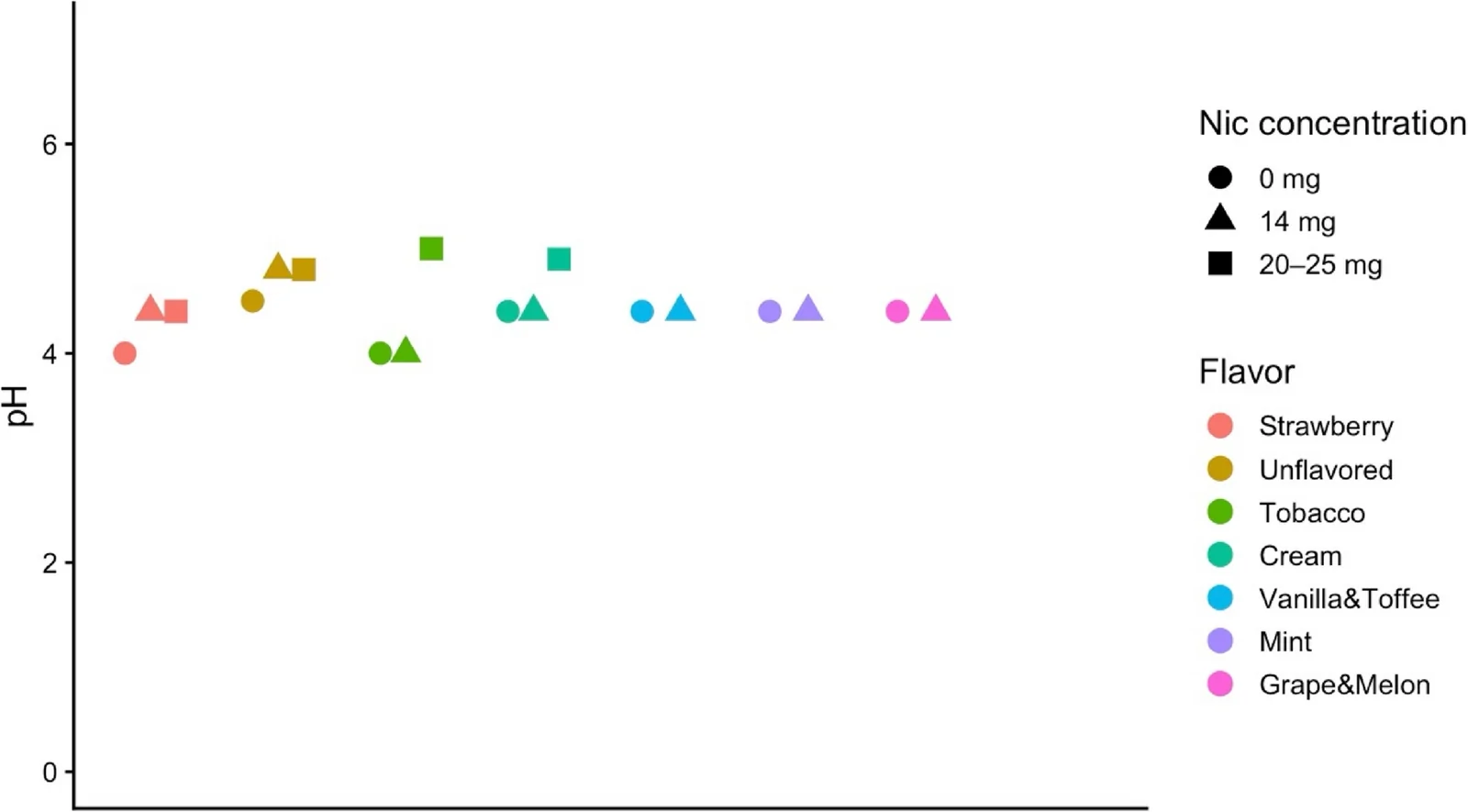
Approximate pH screening values for undiluted e-liquids, measured with semi-quantitative pH indicator strips after 1 minute. The values do not represent titratable acidity, aerosol-extract pH, or the pH of the final bacterial exposure mixture

Semi-quantitative strip screening indicated that the approximate pH of all tested undiluted e-liquids ranged from 4.0 to 5.0 (Fig. 5). This acidic range was consistent across all formulations, including nicotine-free and unflavoured products. No substantial differences in pH were observed between flavour categories or nicotine concentrations.

## Discussion

This study provides preliminary descriptive observations on single-species planktonic growth responses of selected oral bacteria to e-cigarette aerosol extracts, together with an exploratory direct-exposure experiment using undiluted e-liquids and *S. mutans*, an agar diffusion screen for local growth inhibition, and approximate pH screening of undiluted e-liquids. The observed patterns were species- and formulation-dependent under the tested in vitro conditions. Because biological replication was limited and the chemical composition and storage stability of the aerosol extracts were not analytically verified, these findings should not be considered confirmatory evidence of formulation effects. They do not demonstrate bactericidal activity, dysbiosis of cariogenic biofilm, enamel demineralisation, or increased risk of clinical caries.

Previous studies have investigated the effects of e-cigarette liquids, aerosols, flavouring compounds, or nicotine on oral bacteria. However, comparative studies simultaneously evaluating aerosol extracts, undiluted e-liquids, flavour categories, nicotine concentrations, and both cariogenic and commensal oral bacterial species under standardised experimental conditions remain limited. A notable strength of the present study is the standardised comparative evaluation of multiple commercially available e-cigarette formulations across several selected oral bacterial species. While previous studies have often focused on individual flavours, single bacterial species, or isolated compounds, the present design enabled direct comparison of commercial flavour labels, stated nicotine concentrations, and exposure matrices under identical experimental conditions. This comparative approach provides a standardised assessment of species-dependent bacterial responses across multiple commercially available e-cigarette formulations. The inclusion of both aerosol extracts and undiluted e-liquids further enabled comparison between two exposure matrices that may differ substantially in their biological effects. In addition, bacterial growth, antimicrobial activity, and e-liquid acidity were assessed, providing complementary information on the effects of e-cigarette products on oral bacteria. However, the study supports comparative observations at the formulation level but cannot identify the compounds responsible for the observed effects.

Across most tested product–species combinations, mean AUC values observed following exposure to e-cigarette aerosol extracts were lower than the corresponding untreated growth control, although the magnitude and, in some cases, the direction of the observed difference varied by species and formulation. Among the species evaluated, *A. naeslundii* showed the largest descriptive reduction in mean AUC under the tested conditions, whereas smaller changes were observed for several streptococcal species. These preliminary patterns are consistent with previous in vitro studies reporting that e-cigarette liquids or aerosols can suppress bacterial growth, with species-dependent responses [24, 27, 28]. However, it remains difficult to determine how these findings translate to the oral environment in vivo. In the oral cavity, microbial interactions occur within structured biofilms, where competition, cooperation, saliva, the acquired pellicle, nutrient gradients, and host factors influence bacterial survival. Therefore, inhibitory effects observed in single-species planktonic cultures may not directly translate to in vivo conditions. Although simplified, planktonic models remain useful for identifying direct antimicrobial and physicochemical effects prior to investigation in more complex multispecies biofilm systems. Single-species planktonic inhibition may therefore suggest a potential to influence ecological balance under repeated exposure, but it does not show that dysbiosis or community restructuring occurred.

The observations obtained for *S. mutans* require particular consideration in relation to exposure matrix and study design. Although AUC reduction was observed following exposure to aerosol extracts, larger reductions were detected when undiluted e-liquids were directly applied. This finding highlights the importance of considering both exposure matrix and concentration when evaluating the effects of e-cigarette products on oral bacteria. Previous studies have reported that e-cigarette aerosols may suppress commensal bacteria more strongly than *S. mutans*, potentially creating conditions favourable for cariogenic species [24]. The discrepancy between studies may be explained by differences in exposure models (aerosol versus extract), chemical formulation of the e-liquids, and the experimental endpoints assessed (planktonic growth versus biofilm formation). Importantly, planktonic proliferation represents only one aspect of cariogenic potential; adhesion, extracellular matrix production, acidogenicity, aciduricity, and community interactions were not evaluated.

In the present study, no consistent descriptive pattern in bacterial growth was observed between commercial flavoured and unflavoured aerosol extract groups across most species, although flavour-dependent effects were noted for *S. sobrinus*. Previous studies have reported effects associated with particular flavouring agents [22, 26, 27, 29], but the present formulations were not chemically characterised. Such discrepancies likely reflect differences in chemical composition, concentration, and formulation between products, as well as variability in experimental conditions. The observed species-dependent responses may reflect differences in the chemical composition of the tested formulations and the compounds generated during aerosolisation. Future studies integrating chemical characterisation with microbiological and host-response outcomes will be important for identifying the constituents most strongly associated with biological effects.

No consistent descriptive pattern was observed across labelled nicotine concentrations in the aerosol extract experiments. However, when undiluted e-liquids were applied to *S. mutans*, differences between nicotine concentrations were observed, suggesting that both concentration and exposure matrix play critical roles. The use of nicotine salts in the tested formulations may also contribute to these findings, as their physicochemical properties differ from those of freebase nicotine used in earlier studies. These findings should not be interpreted as isolated nicotine effects because nicotine form and the concentrations of co-constituents were not analytically verified. Furthermore, the buffering capacity of PBS used during extract preparation likely attenuated pH-related effects, potentially masking the contribution of nicotine-associated acidity.

The agar diffusion assay differentiated aerosol extracts, which showed no detectable zones, from undiluted e-liquids, which produced zones of growth inhibition for several species. A zone indicates growth inhibition around the disk but does not prove killing of the bacteria. Therefore, the results should be regarded as evidence of growth-inhibitory activity under agar diffusion conditions. In this exploratory assay, several flavoured e-liquids showed larger inhibition zones than the unflavoured formulations. This descriptive pattern may reflect differences in formulation composition; however, it cannot be attributed specifically to flavouring compounds because the products were not chemically characterised and inhibition-zone size is also influenced by viscosity and diffusion. Previous studies have reported antimicrobial properties for certain flavouring chemicals, particularly at higher concentrations, but the present data do not permit attribution of the observed zones to specific constituents.

Species-dependent growth inhibition could, in general, affect ecological balance under repeated exposure if particular species are more susceptible than others. However, the present single-species experiments did not measure community composition or restructuring over time. Thus, ecological implications should be treated as hypotheses for multispecies biofilm studies and not as proof that dysbiosis occurred.

All tested undiluted e-liquids exhibited approximate pH values ranging from 4.0 to 5.0, including nicotine-free and unflavoured formulations. These measurements indicate that the undiluted products were acidic under the screening conditions. However, because pH was assessed semi-quantitatively in the undiluted products and neither titratable acidity nor the pH of aerosol extracts, saliva-buffered exposures, or final bacterial culture mixtures was determined, these measurements should be regarded as physicochemical screening observations and cannot be directly translated into enamel demineralisation or clinical erosive potential. The buffering capacity of saliva and the transient nature of exposure in vivo must also be considered when interpreting these findings.

From a clinical and preventive dentistry perspective, the findings identify hypotheses that merit further testing rather than immediate patient-level conclusions. These findings may be particularly relevant given the increasing prevalence of e-cigarette use among adolescents and young adults. Repeated long-term exposure to acidic and biologically active e-cigarette aerosols may contribute to ecological alterations within the oral biofilm and potentially affect salivary protective functions, including buffering capacity and microbial homeostasis. Although the present findings are exploratory and based on an in vitro model, they suggest that vaping-related exposures could represent an additional environmental factor capable of influencing oral microbial ecology and conditions associated with cariogenic biofilm development over time. Increased awareness among dental professionals regarding the potential oral effects of e-cigarette use may therefore be important in preventive treatment and patient risk assessment.

Several limitations directly constrain the strength of the conclusions. First, the use of two independent single-species planktonic cultures does not reflect the complexity of multispecies oral biofilms. The objective of the present study was not to reproduce the complexity of oral biofilms, but rather to provide a controlled screening model capable of identifying direct species-dependent responses to e-cigarette-derived exposures. Such reductionist models remain valuable as a first step before more complex multispecies and biofilm-based investigations. Nevertheless, caution is warranted when extrapolating these findings to the oral environment, where microbial interactions, host factors, and environmental gradients influence bacterial behaviour. Second, the in vitro model does not account for salivary flow, host immune factors, or repeated exposure patterns found in the oral cavity. Third, the use of PBS for aerosol extraction likely reduced pH-related effects and may have influenced the results. Fourth, the chemical composition of the e-liquids was not analytically characterised, making it difficult to determine which specific compounds contributed to the observed effects. A further limitation is that the chemical composition of the aerosol extracts was not characterised in the present study. Consequently, commercial flavour labels and stated nicotine concentrations are crude grouping variables that cannot support mechanistic attribution to specific constituents. Ongoing investigations involving chemical characterisation of the extracts and host-response models may help clarify the mechanisms underlying these observations. Moreover, chemical stability of the aerosol extracts during storage was not independently evaluated, but storage at − 80 °C was employed to minimise sample degradation. Therefore, the possibility of alterations in volatile, reactive, or poorly water-soluble constituents during storage cannot be excluded. In addition, pH measurements were performed using semi-quantitative indicator strips providing approximate screening values rather than a calibrated digital pH meter, which limited the precision of the measurements. Nevertheless, all tested e-liquids consistently showed acidic pH values within a range relevant to enamel demineralisation. The statistical results should be considered preliminary and descriptive, and specific to the bacterial species and e-cigarette formulations chosen and tested in this study; they are not estimates of effects across the wider range of commercially available products. Finally, the disk diffusion method is dependent on viscosity and diffusion, and accuracy of duplicate testing is limited.

The two additional species, B. dentinum and the Lactobacillus clinical isolate, were included only in the exploratory disk diffusion panel and should not be considered part of the primary growth curve comparison. Future studies should include chemical characterisation of e-cigarette formulations together with multispecies biofilm models under conditions that better reflect the oral environment. Therefore, the present findings should be interpreted as preliminary controlled in vitro observations rather than direct evidence of clinical cariogenic effects.

## Conclusion

Within the limitations of this exploratory single-species in vitro study, descriptive differences in bacterial growth were observed following exposure to e-cigarette aerosol extracts, with the magnitude and direction of these observations varying among species and formulations. Direct exposure of *S. mutans* to undiluted e-liquids was associated with markedly lower mean growth under the tested conditions, and several undiluted formulations produced zones of growth inhibition in the agar diffusion assay. Given the limited biological replication (*n* = 2), these findings should be regarded as preliminary descriptive observations rather than evidence of population-level differences among conditions. Confirmation in adequately replicated experiments incorporating chemical characterisation, multispecies biofilm models, and clinically relevant exposure conditions is required before conclusions regarding oral microbial ecology or caries-related effects can be drawn.

## Supplementary Information

Supplementary Material 1.

## Data Availability

The datasets generated and/or analysed during the current study are available from the corresponding author on reasonable request.
