# Supplementary material for: Preliminary species-dependent growth responses of selected oral bacteria to e-cigarette Liquids and aerosol extracts: a single-species in vitro study
Source: BMC Oral Health. 2026 Oct 6;26:1942. doi: 10.1186/s12903-026-10025-x (PMC13640228; doi:10.1186/s12903-026-10025-x)
Supplement: Supplementary file 1 [file 12903_2026_10025_MOESM1_ESM.docx]

Supplementary file:

**Supp. Figure 1.** Bar plots show area-under-the-curve (AUC) values for each sample and species. Positive controls consistently exhibited the highest AUC values, while all e-liquid formulations reduced growth to varying degrees. (A) *A. naeslundii,* (B) *L. rhamnosus,* (C) *S. mitis,* (D) *S. salivarius,* (E) *S. sanguinis,* (F) *S. sobrinus, (*G) *S. mutans,* and (H) *S. mutans* (100% e-liquids).

**Supp. Figure 2**. AUC values grouped by nicotine level (0 mg/ml, 14 mg/ml, 20–25 mg/ml). (A) *A. naeslundii,* (B) *L. rhamnosus,* (C) *S. mitis,* (D) *S. salivarius,* (E) *S. sanguinis,* (F) *S. sobrinus, (*G) *S. mutans,* and (H) *S. mutans* (100% e-liquids).

**Supp. Figure 3.** Agar plate for one of the bacterial species showing a duplicate of 3 different e-liquid extracts with a central control disk with Chlorhexidine.

**Supp. Table 1.** **Area under the curve (AUC) values for different oral bacterial species following exposure to e-cigarette aerosol extracts. Data are presented as mean ± standard deviation (SD) (n = 6)**

| Group | *L. rhamnosus* | *S. salivarius* | *S. sanguinis* | *S. mitis* | *S. sobrinus* | *A. naeslundii* | *S. mutans* (Extracts) | *S. mutans*  (E-liquid 100%) |
| --- | --- | --- | --- | --- | --- | --- | --- | --- |
| Positive Control | 14.1 ± 5.0 | 23.4 ± 3.1 | 20.3 ± 6.6 | 11.9 ± 3.0 | 23.8 ± 1.1 | 18.5 ± 2.9 | 20.3 ± 2.4 | 12.0 ± 8.1 |
| Cream 0 Nic | 10.8 ± 4.4 | 19.7 ± 2.8 | 17.1 ± 5.9 | 10.3 ± 2.7 | 20.3 ± 2.3 | 12.6 ± 1.0 | 17.1 ± 2.3 | 2.5 ± 0.2 |
| Cream 14 mg Nic | 11.2 ± 5.3 | 20.7 ± 3.6 | 17.6 ± 5.7 | 10.6 ± 2.6 | 21.6 ± 3.5 | 14.1 ± 1.4 | 17.2 ± 2.7 | 2.5 ± 0.1 |
| Cream 25 mg Nic | 11.1 ± 4.5 | 19.5 ± 2.6 | 17.2 ± 5.2 | 10.2 ± 2.7 | 20.3 ± 2.7 | 12.9 ± 0.8 | 16.9 ± 2.2 | 3.1 ± 0.2 |
| Grape&Melon 0 Nic | 12.8 ± 5.9 | 22.8 ± 5.5 | 18.8 ± 7.3 | 11.5 ± 2.5 | 26.2 ± 1.6 | 16.0 ± 0.9 | 18.1 ± 3.5 | 2.3 ± 0.1 |
| Grape&Melon 14 mg Nic | 12.7 ± 5.2 | 22.5 ± 5.4 | 17.6 ± 7.5 | 11.8 ± 2.7 | 25.3 ± 1.8 | 15.3 ± 1.0 | 18.2 ± 3.3 | 3.0 ± 0.2 |
| Mint 0 Nic | 11.7 ± 5.8 | 21.1 ± 4.1 | 17.8 ± 6.5 | 11.5 ± 1.8 | 23.2 ± 2.6 | 14.8 ± 0.5 | 16.9 ± 2.0 | 2.8 ± 0.2 |
| Mint 14 mg Nic | 11.2 ± 4.7 | 20.8 ± 3.8 | 17.6 ± 6.6 | 11.0 ± 2.0 | 22.1 ± 2.5 | 13.9 ± 0.7 | 16.8 ± 2.2 | 3.0 ± 0.2 |
| Strawberry 0 Nic | 11.6 ± 4.9 | 21.1 ± 4.1 | 19.0 ± 5.5 | 10.3 ± 2.5 | 22.2 ± 2.9 | 14.3 ± 1.2 | 16.9 ± 1.7 | 2.7 ± 0.6 |
| Strawberry 14 mg Nic | 11.2 ± 4.4 | 20.5 ± 3.7 | 17.6 ± 5.9 | 11.0 ± 2.5 | 21.4 ± 2.8 | 13.8 ± 1.2 | 17.4 ± 1.9 | 3.3 ± 0.1 |
| Strawberry 25 mg Nic | 11.5 ± 4.9 | 20.9 ± 3.6 | 17.7 ± 6.7 | 10.3 ± 2.9 | 21.8 ± 3.4 | 14.3 ± 1.5 | 17.1 ± 2.1 | 2.4 ± 0.1 |
| Tobacco 0 Nic | 11.4 ± 5.0 | 20.5 ± 3.1 | 17.3 ± 6.1 | 10.0 ± 2.7 | 21.5 ± 3.1 | 13.9 ± 1.5 | 17.2 ± 2.5 | 2.2 ± 0.1 |
| Tobacco 14 mg Nic | 10.9 ± 4.2 | 20.0 ± 2.9 | 17.2 ± 5.4 | 10.0 ± 3.0 | 20.6 ± 2.4 | 12.8 ± 0.6 | 16.8 ± 2.0 | 2.8 ± 0.1 |
| Tobacco 25 mg Nic | 11.0 ± 4.8 | 20.2 ± 4.1 | 17.5 ± 6.4 | 10.2 ± 2.6 | 21.7 ± 3.2 | 14.0 ± 1.5 | 16.8 ± 2.3 | 2.3 ± 0.0 |
| Unflavoured 0 Nic | 11.1 ± 4.1 | 20.5 ± 3.1 | 18.0 ± 4.9 | 10.5 ± 2.7 | 21.3 ± 2.7 | 13.2 ± 1.1 | 17.1 ± 2.1 | 2.3 ± 0.4 |
| Unflavoured 14 mg Nic | 11.2 ± 5.0 | 20.6 ± 3.3 | 17.7 ± 5.7 | 10.1 ± 2.7 | 21.4 ± 3.0 | 14.1 ± 1.6 | 16.5 ± 2.2 | 2.1 ± 0.2 |
| Unflavoured 20 mg Nic | 11.0 ± 4.4 | 20.2 ± 2.7 | 17.7 ± 5.7 | 10.5 ± 2.6 | 20.7 ± 2.2 | 13.1 ± 0.9 | 17.0 ± 2.0 | 2.1 ± 0.2 |
| Vanilla&Toffee 0 Nic | 11.4 ± 5.2 | 20.3 ± 3.6 | 17.3 ± 6.1 | 10.9 ± 2.3 | 21.7 ± 3.2 | 14.1 ± 0.9 | 17.0 ± 2.5 | 2.4 ± 0.1 |
| Vanilla&Toffee 14 mg Nic | 10.9 ± 4.4 | 20.2 ± 3.1 | 17.7 ± 5.1 | 10.3 ± 2.3 | 21.5 ± 3.0 | 13.1 ± 0.6 | 17.1 ± 2.0 | 3.2 ± 0.2 |

**Supp. Table 2. Antibacterial effect (mm) of flavoured e-liquids at different nicotine concentrations against oral bacterial species.**

| **Flavor** | **Nicotine (mg/mL)** |  | | | |  | | | | |
| --- | --- | --- | --- | --- | --- | --- | --- | --- | --- | --- |
|  |  | ***S. mutans*** | ***S. sobrinus*** | ***L. clinical strain*** | ***B. dentinum*** | ***S. mitis*** | ***S. sanguinis*** | ***L. rhamnosus*** | ***S. salivarius*** | ***A. naeslundii*** |
| Strawberry | 0 | 1.75 | 1.25 | 0.5 | 1 | 0 | 1.5 | 1.25 | 0.75 | 1 |
|  | 14 | 3 | 1 | 0.75 | 0.25 | 0.25 | 1.5 | 0.5 | 2 | 1 |
|  | 25 | 1.5 | 1.5 | 1.25 | 0.75 | 0.75 | 2.25 | 2 | 0.5 | 1 |
| Unflavoured | 0 | 0 | 0 | 0 | 0 | 0 | 0 | 0 | 0 | 0 |
|  | 14 | 0 | 0 | 0 | 0 | 0 | 0 | 0.5 | 0 | 0 |
|  | 20 | 0 | 0 | 0 | 0 | 0 | 0 | 0 | 0 | 0 |
| Tobacco | 0 | 0 | 1.25 | 0.5 | 0 | 0 | 0.25 | 0.75 | 0 | 0 |
|  | 14 | 0.5 | 2 | 1 | 1 | 0 | 3.25 | 0.75 | 0 | 0 |
|  | 25 | 0 | 0 | 0.25 | 0.5 | 0 | 0 | 0 | 0 | 0 |
| Cream | 0 | 0 | 0 | 0 | 0 | 0 | 0 | 0.75 | 0.5 | 0.25 |
|  | 14 | 0.5 | 1.5 | 0 | 0 | 0 | 0.5 | 0.25 | 0 | 0 |
|  | 25 | 0 | 1.5 | 0 | 0 | 0 | 6.5 | 0 | 0.25 | 0 |
| Vanilla & Toffee | 0 | 0.5 | 0.5 | 0 | 0 | 0 | 0 | 0.75 | 0 | 0 |
|  | 14 | 0.75 | 1 | 0 | 0.75 | 0 | 3 | 0 | 0.25 | 0 |
| Mint | 0 | 1 | 1.25 | 0.5 | 0.5 | 0.25 | 1.5 | 0.5 | 0.75 | 0 |
|  | 14 | 1.25 | 0.5 | 1.5 | 0.25 | 1.25 | 4 | 1 | 0.5 | 0.75 |
| Grape & Melon | 0 | 1.75 | 0.5 | 0 | 0 | 0 | 0 | 1 | 1 | 0.5 |
|  | 14 | 0.75 | 1.5 | 1.5 | 0 | 1.5 | 1 | 0.25 | 1 | 0.25 |

**Note.** Values represent antibacterial effect (mm). A value of 0 indicates no detectable effect.
